# Synergic Effect of Recycled Carbon Fibers and Microfibrillated Cellulose Gel for Enhancing the Mechanical Properties of Cement-Based Materials

**DOI:** 10.3390/gels9120981

**Published:** 2023-12-14

**Authors:** Matteo Sambucci, Seyed Mostafa Nouri, Sara Taherinezhad Tayebi, Marco Valente

**Affiliations:** 1 Department of Chemical Engineering, Materials, Environment, Sapienza University of Rome, 00184 Rome, Italy; nori.mostafa@gmail.com (S.M.N.); sara.taherinezhadtayebi@uniroma1.it (S.T.T.); marco.valente@uniroma1.it (M.V.); 2 INSTM Reference Laboratory for Engineering of Surface Treatments, UdR Rome, Sapienza University of Rome, 00184 Rome, Italy

**Keywords:** hybrid fiber-reinforced mortar, microfibrillated cellulose gel, recycled carbon fiber, mechanical properties, water absorption, porosity, microstructure

## Abstract

A new hybrid fiber blend containing microfibrillated cellulose (MFC) gel and recycled carbon short fiber (RCSF) was implemented for designing fiber-reinforced cement mortars, to further improve the mechanical properties and enhance the sustainability of cement-based materials. The individual impact of single fibrous fillers as well as the synergistic effect of a hybrid fiber system (MFC + RCSF) were investigated in terms of the rheological properties, mechanical strength, and microstructure of the mortars. The results indicated that the workability of fresh mixtures slightly increased after fiber addition. The fibers incorporated alone improved the materials’ performance in different ways. The addition of RCSF led to improvements of up to 76% in flexural strength and 13% in compression strength for a fiber content of 0.75 wt.%. However, the addition of carbon fibers led to slight deteriorations in terms of porosity and water absorption. On the other hand, the use of MFC induced a less significant growth in terms of mechanical strength (+14% in flexural strength for 0.75 wt.% of cellulose) but greatly improved the microstructural quality of the mortar, significantly reducing its water permeability. Considering the optimum MFC dosage, MFC+RCSF hybrid mixtures showed positive effects on the mechanical properties and microstructure of the mortar, displaying further improvements in strength, while preserving a lower porosity and water absorption than the control mix.

## 1. Introduction

The utilization of concrete has been widely adopted due to its high structural resistance and durability, making it the most widely used material in the construction industry [1,2]. Globally, concrete has been placed as the second most-used substance [3], with three tons per year usage for each person living on earth [4]. Besides its various advantages, concrete has certain mechanical and morphological limitations, including weakness in tension and the presence of micro-cracks and capillaries or micro-capillaries [5]. On the other hand, academia and industry have reported that concrete production can damage our eco-system and have environmental drawbacks. For instance, it is responsible for approximately 9% of worldwide greenhouse gas emissions [6], specifically CO_2_; about 7–8% of the mentioned percentage is the cement implant’s share [7]. Unfortunately, another previously stated environmental hazard of concrete production is that it needs extensive energy [8,9] and water supply [10,11]. Moreover, for producing concrete, a large amount of raw minerals and materials (such as limestone, clay, natural sand, gravel, fibers, and other admixtures) have to be extracted from our planet and therefore consumed [12,13].

To tackle the challenges above, it is crucial to enhance the sustainability of concrete to meet the present and future demand and, to some extent, minimize its environmental effects. The incorporation of fibers like steel, glass, carbon, polymeric fibers, and cellulose inside the concrete matrix, commonly known as fiber-reinforced concrete (FRC), is an approach to boost sustainability. These fibers can be mixed with concrete to improve specific characteristics, from compressive strength and toughness to flexural strength and toughness and abrasion resistance [4]. As a result, fiber-reinforced concrete (FRC) shows superior performance compared to non-reinforced concrete, allowing for a reduction in the amount of concrete required to achieve the same level of performance. Consequently, less concrete needs to be produced, leading to a decrease in the negative environmental impacts associated with concrete. This approach contributes to promoting concrete sustainability [14]. 

Among the fibers used for reinforcement of concrete, carbon fibers (CFs), as a synthetic fiber, have numerous desirable properties, from high thermal and electrical conductivity to excellent chemical stability [15]. Addition of CFs to concrete provides better durability and simultaneously reduces the weight of FRC (because of the low density of CF) [16,17]. Moreover, the elastic modulus of most synthetic fibers integrated into FRC is around 7 GPa, whereas the value of this property for CFs ranges between 21 and 40 GPa. Therefore, the addition of CFs can improve concrete properties significantly [18]. Moreover, their incorporation in concrete assists the cementitious matrix, not only in diminishing shrinkage and cracking, but also in increasing the tensile strength, flexural toughness and strength, impact resistance, and freeze–thaw durability [19,20].

Despite all the mentioned advantages that CFs can provide to concrete, virgin CFs are expensive, and it is not economically acceptable to use them in concrete. The key to solving this issue is using recycled carbon fibers (RCFs) instead of virgin fibers. This has a low cost, and furthermore using them contributes to a circular economy and waste management [21]. On the other hand, surprisingly, the recycling processes, such as pyrolysis, does not affect the mechanical properties. As a result, they have almost all identical properties to virgin CFs after integrating them into concrete [22].

However, it should be noted that the hydrophobicity of CFs results in the agglomeration of the fiber generally and inside the concrete. This phenomenon adversely affects the microstructure and the permeability of the cement paste, especially at high-volume fractions [23]. One approach to tackling this issue is to include a second fiber inside the concrete, which can lessen the drawbacks associated with the primary fiber. This method is called hybridization and hybrid fiber-reinforcement has been developed as an effective technique to combine the positive qualities of various fibers [5].

It has been proven that cellulose, a bio-based substance derived from plants, can be utilized for the hybridization process and act as a second fiber in conjunction with CFs. In this regard, through hybridization of cellulose and CF, the concrete will benefit from both, and this can also make up for the loss of properties found in CF-reinforced concrete. Low cost, renewability, wide availability, lightweight, and biodegradability are several intrinsic properties reported for cellulose [24,25,26]. It has been found that shrinkage cracking and water absorption rates can be reduced by incorporating cellulose fibers into concrete. Nevertheless, the freeze–thaw performance, toughness, and fire resistance become upgraded with this action. Furthermore, cellulose fibers can hinder crack generation and propagation, as well as permeability; on the other hand, they can protect embedded steel rebars, and improve frost and impact resistance [27]. Notably, for the sake of a better function, cellulose fibers must be treated before use. When untreated natural fibers are employed and inserted into concrete, specific problems such as low durability and poor adhesion to the matrix, and inconsistent properties, are likely to occurr [28]. In addition, if cellulose fibers without treatment are added to the cement matrix, they are more susceptible to degradation in the alkaline environment of concrete [29]. Therefore, in the recent past, instead of cellulose fibers, a second generation known as cellulose nanofibrils (NFCs)/or microfibrillated cellulose (MFC) have been introduced and investigated [30]. These products with an average nanometric-micrometric size range exhibit superior characteristics and properties compared to natural cellulose fibers [31].

This paper investigates the combined effects of MFC gel and recycled carbon short fiber (RCSF) in concrete. While the individual benefits of MFC gel and RCSF have been studied, their synergistic effects have yet to be extensively explored. Hence, three families of specimens were prepared. The first family contained three different percentages of MFC gel (0.5 wt.%, 0.75 wt.%, and 1 wt.% with respect to water). The second family contained three different percentages of RCSF (0.5 wt.%, 0.75 wt.%, and 1 wt.% with respect to cement). Finally, for the third family, the optimum percentage of MFC gel was combined with three percentages of RCSF (0.5 wt.%, 0.75 wt.%, and 1 wt.% with respect to cement). Subsequently, the sample microstructure, mechanical and rheological behavior, porosity, and water absorption rate were evaluated. The aim of this study is to provide a valuable insight into optimizing concrete mix design to develop a more sustainable and high-performance construction material. The findings of this work will pave the way for improvements in concrete technology and facilitate the development of efficient and durable infrastructure materials.

## 2. Results and Discussion

### 2.1. Slump Test

**Figure 1:**
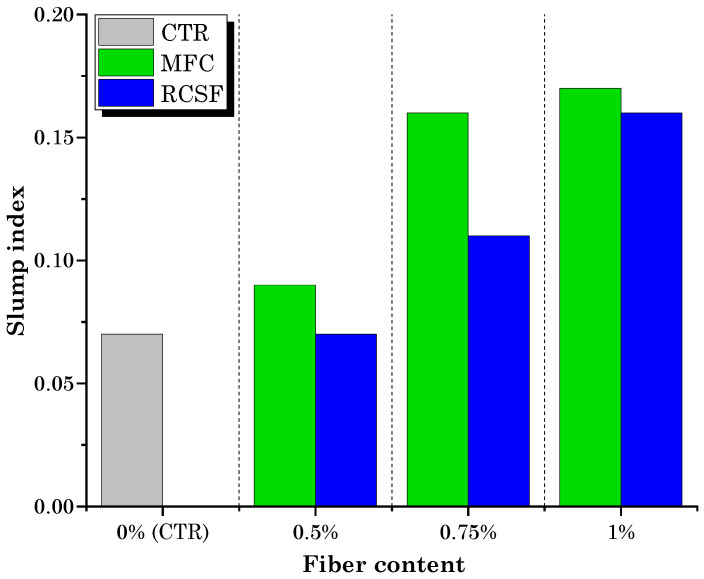
Slump index for mixtures containing MFC and RCSF.

Figure 1 presents the results of the slump test for the control mixture (CTR) and mixtures containing different dosages of MFC gel and RCSF. The data clearly indicate that an increase in the dosage of both MFC gel and RCSF corresponded to an improvement in workability.

**Figure 2:**
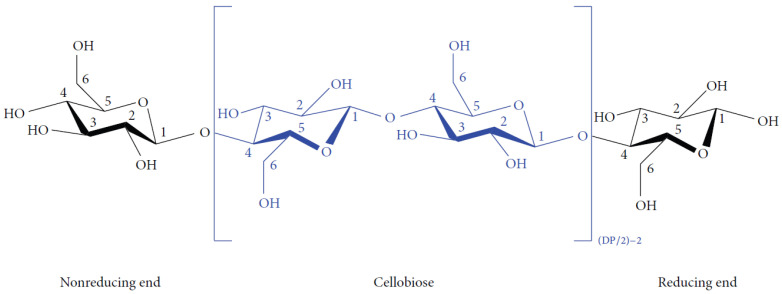
Chemical structure of MFC [32].

In case of mortars containing MFC gel, the increased flowability can be attributed to the high-water content of the MFC gel, which contained 98 wt.% water. The addition of MFC gel to the concrete mixture elevated the overall water content, consequently enhancing the workability of the concrete as the dosage of MFC gel increased [32]. Another possible reason for this phenomenon may be related to the significant viscosity-modifying effect introduced by the MFC. This effect arises from the hydrophilic nature of cellulose-based fibers, characterized by an abundance of surface hydroxyl (OH−) groups, as shown in Figure 2 [33]. The viscosity modifying effect of MFC also results from the flexible and nanometric fibrillar structure of filaments, leading to the creation of nanoscale fibril networks [34]. As a result of this viscosity-modifying effect introduced by the addition of MFC gel, the viscosity buildup was reduced, leading to an increase in the flowability of the MFC gel-reinforced concrete [35,36]. According to Yadykova and Ilyin [37], cellulose microfibrils adsorb on the surface of cement and sand particles, thereby reducing the interaction between them, as the interaction between cellulose particles, as organic matter, is weaker than between two inorganic particles with high surface energy. This interfacial adsorption allows obtaining Pickering emulsions, reducing the viscosity of the cementitious mixture.

On the other hand, when examining carbon-reinforced mortars, the observed enhanced workability can be attributed to two key factors: the hydrophobic nature of the carbon fibers and their presence within the mixture in agglomerated form. In these compositions, the carbon fibers predominantly existed in an agglomerated state within the matrix. The hydrophobic characteristics of the carbon fibers induced a redistribution of water within the mixture, favoring areas that did not contain these agglomerated fibers. As a result, water became more readily available in localized regions of the mixture that were distant from the fibers. Consequently, this phenomenon led to an overall improvement in the workability of the mixture.

### 2.2. Three-Point Flexural Test

**Figure 3:**
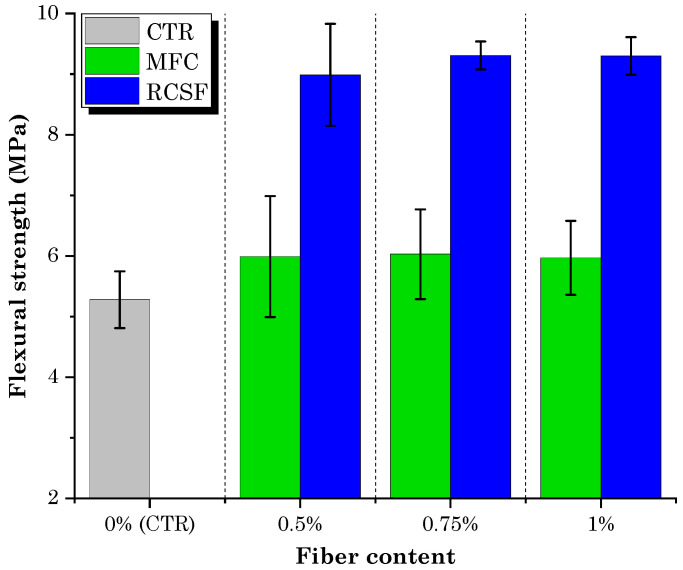
Flexural strength test results for mixtures containing RCSF and MFC gel.

The flexural test results for concrete samples containing different dosages of RCSF and MFC gel are presented in Figure 3.

It is readily apparent that the flexural strength of concrete mixtures containing all dosages of RCSF and MFC gel surpassed that of the control mix (CTR). This observation underscores the positive impact of incorporating RCSF and MFC gel on the flexural strength of concrete.

**Figure 4:**
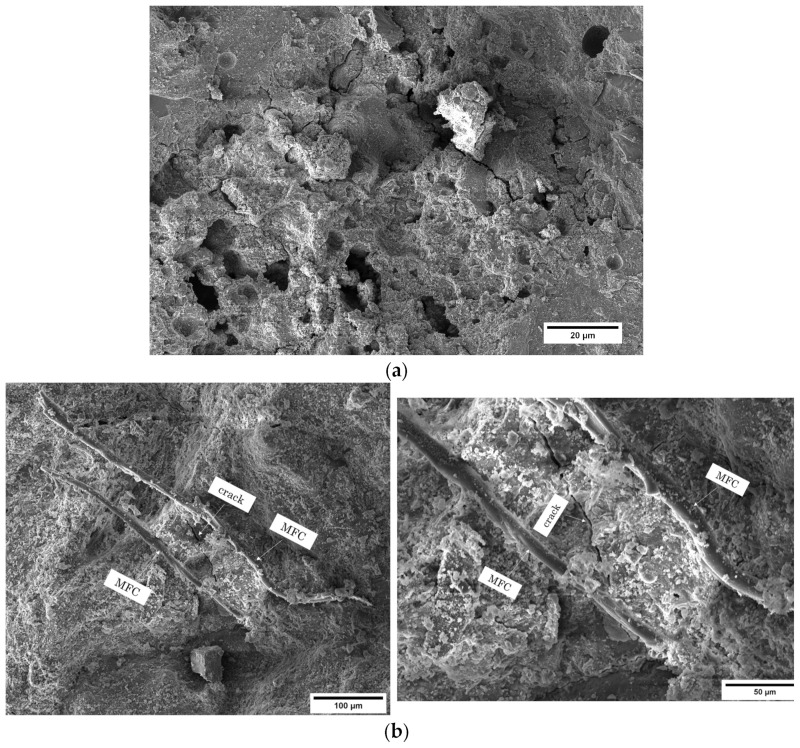
SEM images of (**a**) neat mortar sample and (**b**) concrete sample containing MFC, showing the crack-arresting effect of cellulose fibrils and strong fibril–cement interaction.

A closer look at the results of the MFC gel-reinforced samples shows that the 0.75M sample exhibited the highest improvement in flexural strength, with a 14.2% increase compared to CTR. For the 0.5M and 1M samples, the increments in flexural strength were 13.5% and 13%, respectively. The enhanced flexural strength of concrete achieved through the inclusion of MFC gel can be attributed to several influential factors:

1. The hygroscopic nature of MFC influences the hydration kinetics of concrete [36,38]. To elaborate, the polar hydroxyl groups within the MFC structure (as depicted in Figure 2) establish hydrogen bonds with water molecules. This interaction leads to the absorption of water by MFC. During the later stages of concrete hydration, MFC releases the entrapped water into the cementitious matrix, thereby facilitating the ongoing hydration of previously unreacted cement particles. This phenomenon ultimately enhances the mechanical performance of the concrete [39,40,41,42];
2. Cellulose fibrils may induce a bridging effect that impedes crack propagation, elevating the energy threshold necessary for crack advancement. As a result, this mechanism enhances the overall flexural strength of the concrete. This crack-arresting effect of cellulose fibrils is visually evident in SEM images of samples containing MFC gel (Figure 4) [38,43,44]. Figure 4a shows the crack pattern of a neat concrete sample (no fibers). Figure 4b displays the crack suppression effect of MFC.
3. The significant specific surface area of MFC results in a substantial number of hydrogen bonds forming between the surface hydroxyl groups on the fibrils and the cement matrix. This phenomenon fosters a strong interfacial adhesion between cellulose fibrils and the matrix [45,46], a fact corroborated by the SEM images of MFC-incorporated concrete (Figure 4b above) in this study. This robust bonding enhances the capacity of the fibrils to effectively transmit stress within the concrete matrix when subjected to flexural loading conditions. Consequently, the stress distribution becomes more uniform throughout the concrete matrix, ultimately leading to an elevated flexural strength [47].

Nevertheless, it is important to highlight that elevating the MFC gel dosage from 0.75 wt.% to 1 wt.% did not result in a subsequent increase in flexural strength. The inability to achieve an additional improvement in flexural performance can be attributed to fibril aggregation due to inadequate dispersion at higher dosages. This led to the accumulation of clumps within the mortar matrix. In areas where these clumps form, there is a lack of continuity in the cementitious material, potentially leading to stress concentration and, consequently, premature specimen failure [38,48]. 

**Figure 5:**
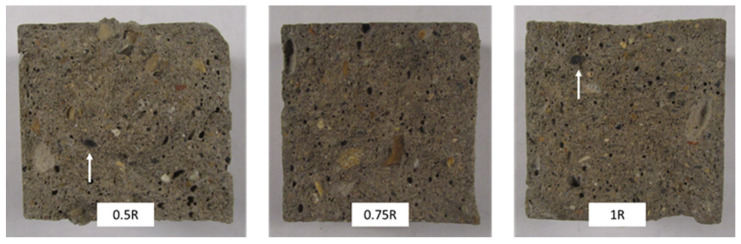
Fractography of the specimens containing RCSF. Arrows indicate the agglomerated CFs within the matrix.

In the case of the mixtures containing RCSF, the improvements in flexural strength exceeded the enhancements seen in the MFC-incorporated samples. The incorporation of RCSF treated with nanoclay at concentrations of 0.5 wt.%, 0.75 wt.%, and 1 wt.% resulted in flexural strength enhancements of 70.5%, 76.3%, and 76.1%, respectively, in comparison to the CTR sample. As discussed in previous work by Sambucci et al. [49], the treatment of carbon fiber with nanoclay results in superior deagglomeration and enhances fiber hydrophilicity. These two factors promote more effective fiber dispersion and mitigate matrix porosity, consequently elevating flexural strength. Moreover, the slight reduction in flexural strength observed when increasing the RCSF dosage from 0.75 wt.% to 1 wt.% can be attributed to the challenges encountered in achieving uniform fiber dispersion at higher concentrations, resulting in the presence of fiber agglomerates within the concrete matrix, which hindered further increases in flexural strength. Observation of the fracture surface (Figure 5) additionally verified the existence of a limited number of clustered fiber fragments within the cement matrix.

**Figure 6:**
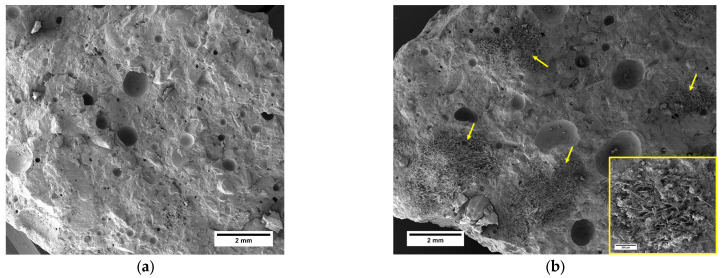
SEM micrographs of (**a**) neat sample and (**b**) RCSF-reinforced mortar displaying the agglomeration of carbon fibers in the matrix (yellow arrows).

The identification of agglomerated carbon fibers in the cementitious matrix is clearly highlighted in the fractured surface SEM micrographs in Figure 6. The microstructure of a neat cementitious sample (Figure 6a) is compared to that of RCSF-reinforced mortar (Figure 6b), displaying carbon fiber clusters (identified by the yellow arrows) embedded in the matrix.

**Figure 7:**
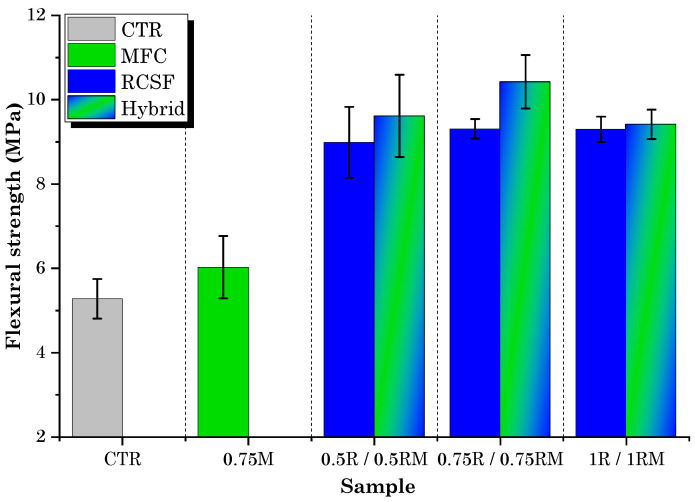
Flexural strength test results for hybrid samples and comparison with RCSF-containing, 0.75M, and CTR samples.

The last category of samples in the experiment, which were prepared with the optimal dosage of MFC gel (0.75 wt.%) based on mechanical test results, along with three different dosages of RCSF (0.5 wt.%, 0.75 wt.%, and 1 wt.%), demonstrated even further improvements in flexural strength for two of the three RCSF dosages (0.5 wt.% and 0.75 wt.%) compared to samples containing RCSF alone. As shown in Figure 7, the flexural strength of the 0.5RM and 0.75RM samples increased by 1.6% and 8.5%, respectively, when compared to the 0.5R and 0.75R samples. Conversely, the flexural strength of the 1RM sample decreased by 4% compared to the 1R sample.

It appears that MFC and RCSF each contributed to increasing the flexural strength in distinct ways. MFC gel, as explained previously, enhances flexural strength through internal curing and matrix bridging, while RCSF contributes to concrete flexural strength by controlling cracks and increasing ductility. Given that MFC’s matrix bridging capability is limited, likely due to its short length compared to RCSF [50], this effect can be neglected in increasing the flexural strength of hybrid samples. Therefore, it can be said that MFC primarily enhances flexural strength through internal curing, while RCSF contributes by controlling cracks and increasing ductility. Consequently, the flexural strength of hybrid samples containing both MFC gel and RCSF exceeds that of samples containing only one of these components. However, in the case of the 1RM sample, the high amount of fibers and their aggregation in the matrix potentially led to a slight decrease in flexural strength compared to the 1R sample.

### 2.3. Compression Test

**Figure 8:**
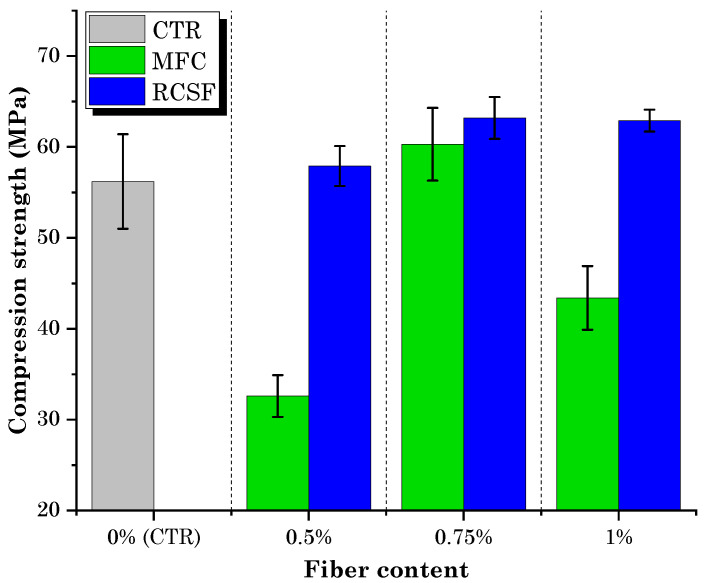
Compressive strength test results for mixtures containing MFC gel and RCSF.

The compression test results for concrete samples containing varying dosages of MFC gel and RCSF are presented in Figure 8.

It is evident that the compressive strength of the concrete mixtures containing RCSF at all dosages exceeded that of the control mixture (CTR). However, the influence of MFC gel on the compressive strength of concrete displayed a non-monotonic effect, depending on the dosage.

On closer inspection of the concrete samples containing MFC gel, it becomes apparent that, compared with the control mixture (CTR), they exhibited a marginal increase (7.4% for 0.75M) or a decrease (−42% for 0.5M and −23% for 1M) in their compressive strength. It is worth noting that the 0.75M sample, which exhibited the highest flexural strength among the MFC gel-incorporated specimens, also showed enhanced compressive strength. This improvement at the 0.75 wt.% MFC gel dosage can be attributed to the hygroscopic characteristic of MFC, as previously explained, which can improve the hydration of concrete [36,38]. Conversely, the decrease in compressive strength at dosages of 0.5 wt.% and 1 wt.% of MFC gel can be attributed to the air entrainment (closed porosity) caused by the addition of MFC to the concrete [48]. Hisseine et al. [48] noted that increasing the dosage of MFC leads to an increase in the air content of the concrete. These air voids serve as weak points in the concrete structure. Under stress, these voids can create stress concentrations, making the concrete more prone to cracking and reducing its overall strength [51]. Another possible reason for the reduction in compression strength at higher dosages (1 wt.% of MFC gel) could be the agglomeration of fibrils due to inadequate dispersion [52].

On the other hand, in the case of samples incorporating RCSF, the compressive strength of the 0.5R, 0.75R, and 1R samples increased by 3.2%, 13%, and 11.9%, respectively. These findings align with the flexural strength results, as both analyses exhibited enhanced strength, and the optimal dosage for both tests was 0.75 wt.%. However, the improvements in flexural strength were more pronounced, as the contribution of fibers became more significant under increased flexural loads [53]. As mentioned in a previous work [49], this increase is attributed to the role of nanoclay in ensuring proper dispersion and compatibilization of RCSF, thereby enhancing the compressive strength due to the higher stiffness of carbon fibers compared to the cement matrix.

**Figure 9:**
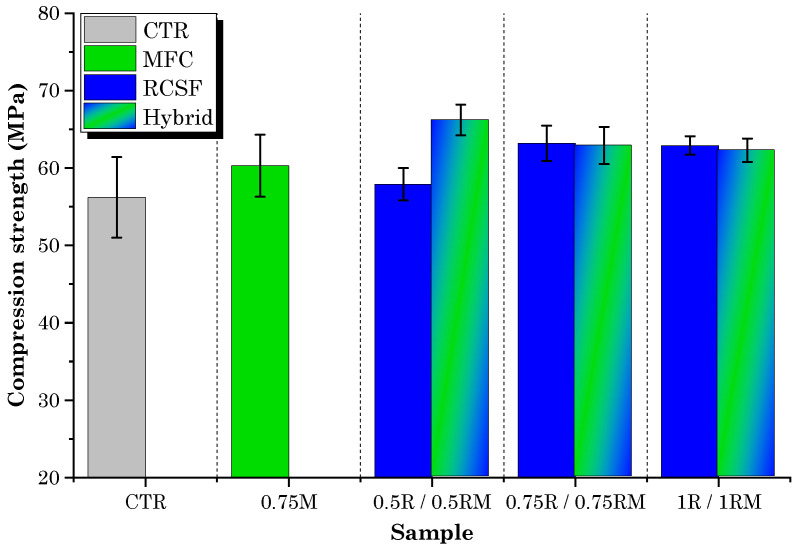
Compressive strength test results for hybrid samples and comparison with RCSF-containing, 0.75M, and control samples.

Figure 9 displays the results of compression testing conducted on hybrid samples, accompanied by a comparative analysis with samples containing RCSF and 0.75M. The graph indicates a slight decrease in compression strength, with reductions of 0.4% and 0.8% observed at dosages of 0.75 wt.% and 1 wt.%, respectively, when compared to samples containing RCSF alone. However, for the 0.5RM sample, the compression strength exhibited a 14.2% increase in comparison to the 0.5R samples. It is worth highlighting that, despite these reductions, all hybrid samples still exhibited higher compression strengths than the CTR samples and the mixture containing 0.75 wt.% MFC gel exclusively. 

### 2.4. Water Absorption and Porosity

#### 2.4.1. Water Absorption

**Figure 10:**
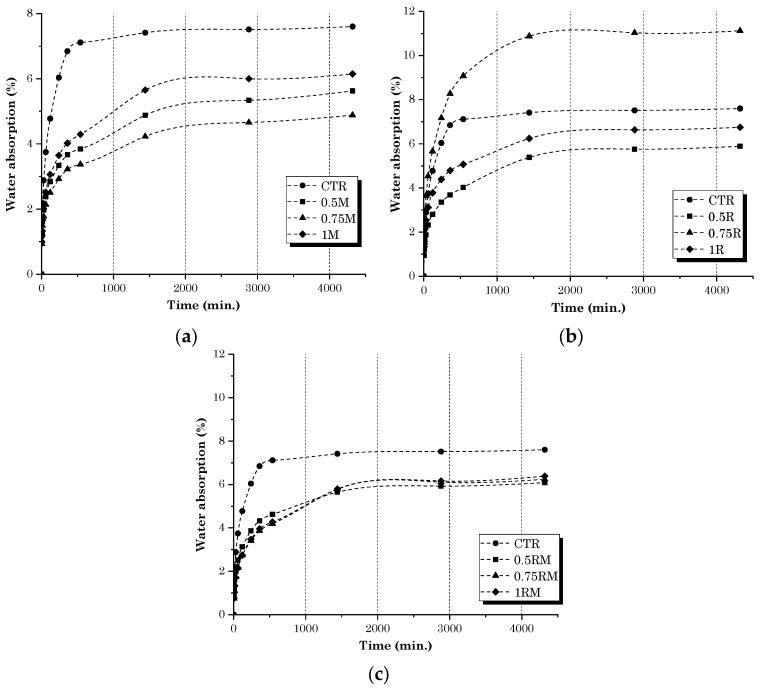
WA results for samples containing (**a**) MFC gel, (**b**) RCSF, and (**c**) hybrid samples.

The water absorption (WA) results for cementitious samples containing different concentrations of MFC gel and RCSF are illustrated in Figure 10a–c, respectively. Apparently, the increase in MFC gel content in the concrete mixture tended to decrease WA with respect to the CTR concrete mix (Figure 10a). This trend could have been due to the hydrophilic characteristics of the gel [53]. Despite the above fact, samples containing 0.75 wt.% MFC gel possessed the lowest WA. On the other hand, samples with 0.75 wt.% cellulose concentration demonstrated superior mechanical performance, correlated with having the best microstructure and less permeable voids. Therefore, the results obtained from the mechanical and WA measurements are aligned. Figure 10b shows that adding RCSF to the cementitious matrix reduced the WA, which differs from the previous research and other works [54,55]. This observation could be related to the nanoclay that was utilized to deagglomerate the RCSF in the matrix. It is worth mentioning that, since clay is a pozzolanic material, the voids and porosity will be less, and the WA will drop correspondingly [56]. Therefore, samples that contained RCSF + nano clay at 1 wt.% dosage demonstrated a maximal effect and minimized the impact of RCSF incorporation. The mixture incorporating the lowest fiber dosage (0.5 wt.%) displayed the lowest water permeability because of reduced carbon contents, the fiber dispersion was more accessible, and there was a minor effect of carbon cluster formation within the concrete’s microstructure. However, the cement composite with 0.75 wt.% RCSF showed higher porosity and absorption rates, which can be attributed to the nanoclay dosage for this composition being insufficient to improve these properties. For hybrid samples (Figure 10c), it is worth mentioning that the addition of MFC gel assisted the reduction in permeability induced by the presence of carbon fibers. All hybrid mixtures showed very similar sorption rates, regardless of RCSF content and significantly below the permeability of the control sample. This finding may demonstrate a synergistic action of the two types of addition to concrete: an increase in mechanical strength due to the carbon fibers, and an improvement in permeability resistance due to the cellulose.

#### 2.4.2. Porosity

**Figure 11:**
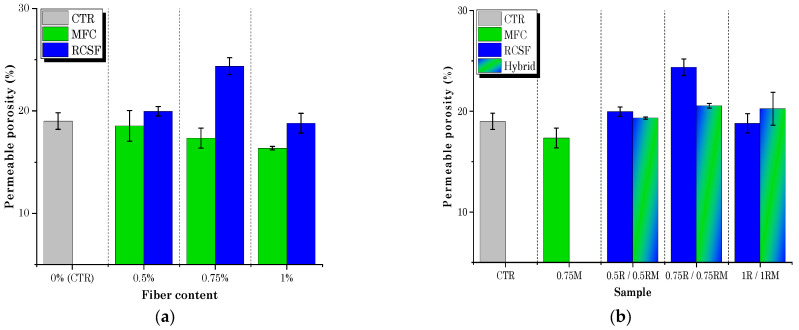
Porosity results for samples containing (**a**) MFC gel and RCSF, and (**b**) hybrid specimens.

**Figure 12:**
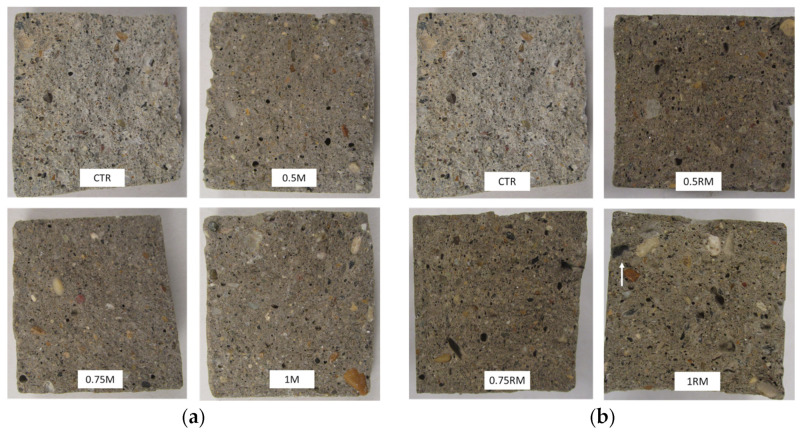
Fracture surface images of the MFC gel (**a**) and the hybrid samples (**b**). White arrow identifies the carbon fiber agglomeration.

Figure 11a represents the porosity results of the cement-based materials in which MFC gel and RCSF were incorporated in the matrix at various dosages. Evidently, the porosity of the specimens decreased as the MFC gel content increased (Figure 11a). This effect of MFC gel could be attributed to the cellulose’s high water retention characteristics. MFC gel prolongs the setting time of cement-based materials and hydrates less hydrated cement particles [54,55]. Therefore, the microstructure of the CTR is refined, and fewer pores are formed [54,55]. Concurrently, the fracture surface images of the concrete samples (See Figure 12a) supported our findings and showed that the concrete mixtures containing MFC gel were more compact than the CTR. Interestingly, as specified in the mechanical properties’ sections, cellulose affected our concrete mixtures from a morphological and microstructural point of view, which increased the flexural and compression strength. In addition, the most significant pore reduction was achieved by integrating 0.75 wt.% MFC gel in the cementitious matrix. Previously, many researchers have reported similar outcomes for the addition of cellulosic components to cement-based composites [31]. 

On the other hand, Figure 11a illustrates that, as a greater RCSF portion was added to the concrete-based composites, the porosity rose. Previously, other scholars described that, due to RCSFs’ hydrophobicity and tendency to agglomerate, the permeable porosity increases accordingly [18,56,57]. Although nanoclay was employed to de-agglomerate the carbon fiber clusters, the porosity of the cementitious composite specimens with 0.5 wt.% and 0.75 wt.% RCSF, compared to the CTR, was slightly elevated. As a result, the amount of nanoclay in these two samples, especially for 0.75 wt.% RCSF, was inadequate to break all the clusters. The porosity changes for the sample reinforced with 1 wt.% RCSF was negligible, and its value was more or less similar to the CTR admixture. Moreover, it is perceptible in the fracture surface images that the RCSF-concrete mixtures had a porous microstructure compared to the CTR. These morphological defects might have influenced the mechanical performance of the specimens. Fortunately, for the RCSF reinforced-concrete mixtures, a higher fiber content had no negative impact on the flexural or compression strength, due to the fibers’ intrinsically superior mechanical properties [56,58]. Other academics have supported these results [58].

Figure 11b depicts the porosity analysis results of the hybrid cementitious composites. As shown in the histograms, incorporating 0.75 wt.% MFC gel content in the samples reinforced with RCSF balanced the number of pores inside the hybrid samples’ matrix, making the microstructure of the samples better (Figure 12b) and preserving their excellent mechanical performance. Therefore, it is noteworthy that, in this research project and for the first time, MFC gel and RCSF had a synergistic effect when combined in hybrid concrete matrixes, and the porosity value was similar to the CTR.

## 3. Conclusions

This manuscript explored the effect of the integration of microfibrillated cellulose (MFC) gel and recycled carbon short fiber (RCSF) in concrete-based composites, investigating their potential synergy to improve the mechanical and microstructural characteristics of cement-based mortars. The conclusions can be summarized as follows:

- The slump index was elevated as the MFC gel and RCSF ratio increased in the concrete composite formulations, demonstrating a slightly improvement in workability following fiber addition;
- Integrating more MFC gel and a higher RCSF content in the cementitious matrix led to an increment in the flexural strength for all dosages. Samples containing 0.75 wt.% MFC gel and 0.75 wt.% RCSF possessed the best flexural strength. Thanks to the effect of MFC gel content in microstructure improvement, in hybrid specimens, the concrete mixtures with 0.5 wt.% and 0.75 wt.% RCSF exhibited superior flexural strength compared to the samples with the same amount of RCSF without MFC gel;
- The compression property analysis revealed that adding 0.75 wt.% MFC gel inside the cementitious matrix resulted in more satisfactory compression strength for than the CTR sample. Despite this, for specimens reinforced with RCSF, higher compression strength was acquired when a higher RCSF content was incorporated. In addition, like the flexural property evaluation, the MFC gel helped the 0.5 wt.% RCSF-hybrid concrete mix offer slightly greater compression strength in contrast to the CTR and the 0.5 wt.% RCSF dosage alone;
- The water absorption capability of the MFC gel samples decreased as a higher ratio of gel was integrated, because of the hydrophilicity of the cellulose gel. Nonetheless, as a higher RCSF percentage was introduced within the cementitious composites, plenty of carbon fiber agglomerations emerged and the WA value increased. For the hybrid mix, the 0.75 wt.% addition of MFC gel improved the results, and the WA was reduced, contrary to the CTR and specimens reinforced with RCSF;
- The porosity results were aligned with the WA, verifying the MFC gel’s high-water retention properties, the hydrophobicity of RCSFs, and their tendency to create clusters.

Ultimately this research has demonstrated the possibility of engineering eco-friendly cementitious mortars with waste materials (RCSF) or bio-based additives (MFC), to obtain specific improvements by exploiting the synergy between two fibers. Future works will be dedicated to implementing the optimized composition of these mixtures in concrete applications. Fiber-reinforced mortar can be integrated as a high-performance face-skin for sandwich-concrete configurations for lightweight construction applications. In addition, fibrous fillers are well suited for designing mortars for additive manufacturing processes. This could take advantage of the synergistic effect between MFC and RCSF to mitigate some critical issues in additive fabrication, including mechanical weakness from the interlayer adhesion, porosity due to the pumping phase, and durability, without compromising the extrusion of the material.

## 4. Materials and Methods

### 4.1. MFC Gel

**Figure 13:**
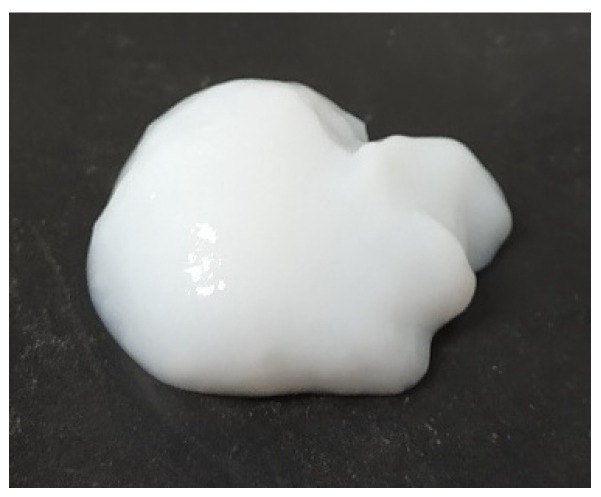
MFC gel.

The MFC gel (Figure 13) used in our experiment was Exilva F 01-L, a product manufactured by Borregaard (Sarpsborg, Norway). It comprises 2 wt.% cellulose fibers and 98 wt.% water. According to the company declaration, this bio-based material is composed of 100% natural MFC, which is pre-activated, odorless, and multifunctional. In addition, this MFC is sustainable and possesses a high aspect ratio, consisting of fibers with nanoscale lateral dimensions and lengths up to micron scale [59]. The use of a cellulose-based product dispersed in an aqueous suspension (gel), rather than the use of a dry filler, prevents the irreversible agglomeration of the fibrils, due to establishment of additional hydrogen bonds between the amorphous regions of cellulose during drying [60], assisting more homogeneous dispersion in the cement matrix.

To prepare the MFC for use in concrete, the determined amount of gel (0.5 wt.%, 0.75 wt.%, and 1 wt.% by weight with respect to the mixing water for each formulation) was initially mixed with 40 g of water in a beaker, stirring at 400 rpm for 5 min. The beaker was then placed in an Elmasonic S30H ultrasound mixer and subjected to 5 min of mixing at 30 °C. After adding the rest of the water required for concrete preparation (in accordance with the mix proportions reported below), the mixture was further stirred for 30 s. 

The surface morphology and chemical composition of the MFC particles were analyzed using a Tescan MIRA 3 (Tescan, Brno, Czech Republic) scanning electron microscope (SEM) equipped with an Energy Dispersive X-ray (EDX) analyzer (Edax, Mahwah, NJ, USA). Prior to analysis, the MFC gel was dried in an oven at 100 °C for one hour, to obtain a solid form. Subsequently, it was gold-coated using an Edwards sputter coater S150B (Edwards Ltd., Burgess Hill, UK).

**Figure 14:**
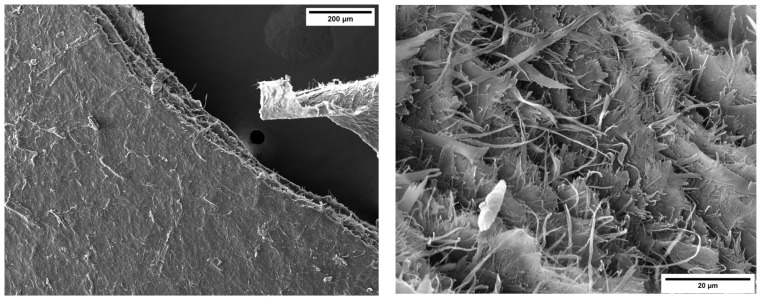
SEM images of the dried form of MFC with different magnifications: 1 kX and 7.5 kX.

As shown in Figure 14, the fibrils appeared compacted within a cellulosic solid matrix forming after the drying process. The material consisted of an entangled network of micrometer/submicrometer-sized irregular and thread-like fibrils having diameters ranging from 500 nm to 2.5 μm.

**Figure 15:**
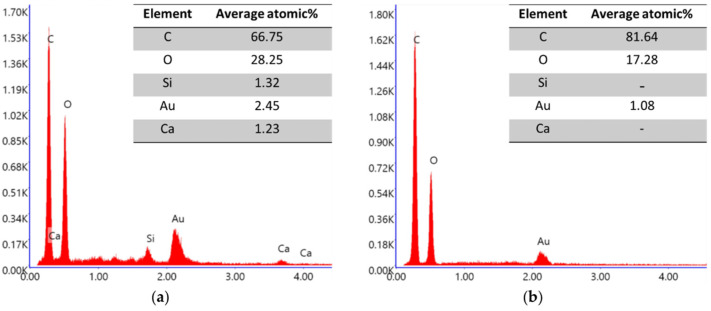
EDX analysis of dried MFC gel. (**a**) Average atomic composition of three EDX measurements of the matrix, (**b**) average atomic composition of three EDX scans of fibrils.

The EDX analysis (Figure 15) confirmed that the fibers primarily consisted of carbon, as expected. Regarding the matrix, the average values indicated that carbon constituted the majority (66.75%) of the composition. Additionally, there were minor percentages of calcium (Ca) (1.23%) and silicon (Si) (1.32%), probably acting as elements to stabilize the fiber gel suspension. However, due to their low concentrations, the presence of silicon and calcium in the matrix was expected to have minimal impact on the properties of the concrete.

### 4.2. RCSF

RCSF is a byproduct obtained from the processing of pyrolyzed carbon fiber to produce woven/non-woven fabrics. The RCSF used in this study was supplied by the Carbon Task company (Biella, Italy). The density of RCSF was determined using the hydrostatic balance method, employing a Mettler Toledo instrument (Mettler Toledo, Columbus, OH, USA), and was found to be 1697 kg/m^3^. Three measurements were taken for density, and the average value was calculated. The surface morphology and dimensions of RCSF were examined using the same instrument that was used for MFC, without any pretreatment because carbon material is already conductive. 

**Figure 16:**
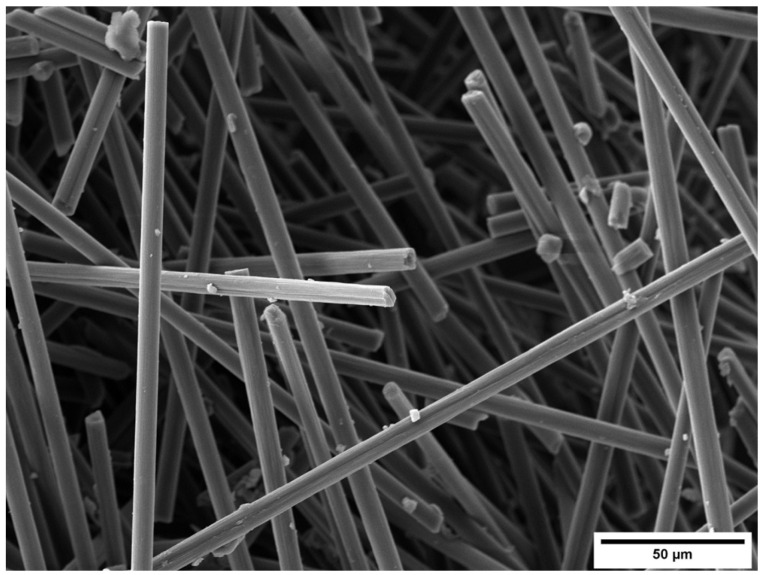
SEM image of RCSF.

Figure 16 displays the results of the SEM observation. It is evident from the images that carbon fibers were long and exhibited regular cylindrical shapes and smooth surfaces. The diameter and length were measured through SEM imaging analysis, yielding an average value of 6.6 µm and 550 µm, respectively. 

Incorporating RCSF into concrete involved an initial treatment process with a commercial Attapulgite nanoclay (ANC), supplied by Lawrence Industries Ltd. (Tamworth, UK), following the procedure developed by Sambucci et al. [49]. According to the data provided by the supplier and the characterization performed in Ref. [49], ANC has a bulk density of 0.769 g/cm^3^, hydrodynamic diameters from 0.75 μm to 3.38 μm, and maximum permitted residue of 45%. The objective was to effectively deagglomerate and disperse the fibers and enhance their compatibility with the cementitious matrix, to deagglomerate the fibers and increase the compatibility between the fibers and cementitious matrix. In this method, first, the required mass of RCSF and water for preparing the concrete mix were magnetically stirred together for 1 min at 500 rpm, then nanoclay with an equal mass with the RCSF was added, and magnetic stirring at 500 rpm was continued for an additional 30 min.

### 4.3. Specimen Preparation

The base cementitious formulation (CTR) consisted of Type-I cement (strength grade of 42.5 R in accordance with UNI EN 197-1 standard [61] and density of 3280 kg/m^3^), 0–1 mm fine river sand (density of 2620 kg/m^3^), and tap water, and the quantities of all these materials remained consistent in all formulations. Starting from the CTR mix, three families of formulations with a constant water/(cement + nanoclay) ratio of 0.42 were designed. The first one contained three different dosages of MFC gel, the second contained three different dosages of RCSF, and the third group included the optimal dosage of MFC gel (determined based on mechanical test results) along with three different dosages of RCSF. Table 1 provides the composition of the mixture constituents used for sample preparation. The specified amounts of materials were sufficient to prepare three specimens with dimensions of 160 mm × 40 mm × 40 mm.

It is important to note that the dosage of MFC gel was based on the weight of water, while the dosage of RCSF was based on the weight of cement. This distinction is because MFC gel is in an aqueous form, whereas RCSF is in a solid form.

For each mixture, three samples were cast in prismatic molds, to obtain beams with a length of 160 mm, a width of 40 mm, and a thickness of 40 mm.

**Figure 17:**
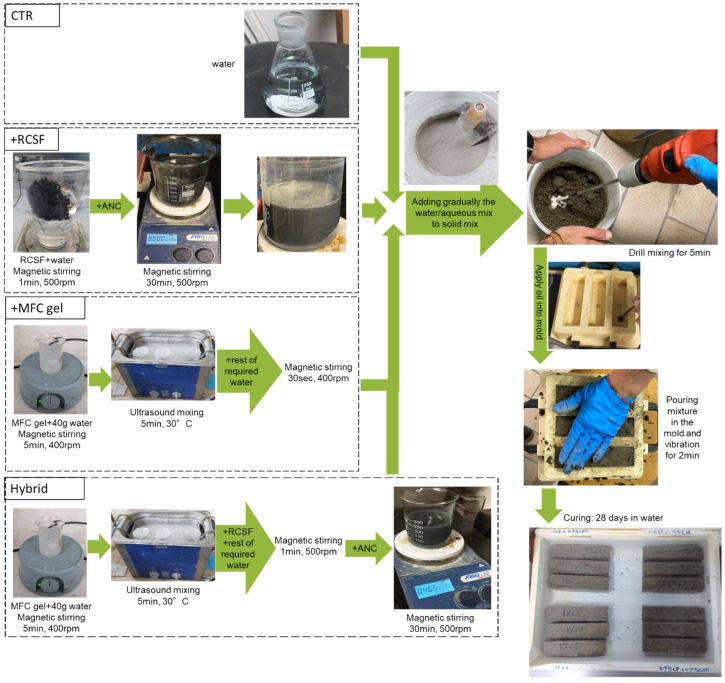
Procedure for preparation of cement mixtures.

Figure 17 displays the procedure for preparation of the different types of samples. In which, for the control sample, only water was mixed with a solid mix, while for samples containing RCSF, fibers was dispersed in a nanoclay slurry, as explained in Section 4.2, then the slurry was added to solid mix; and for samples containing MFC gel, the method described in Section 4.1 was implemented to disperse the fibrils inside water, and after that the prepared aqueous mix along with the rest of required water was added to the solid mix. For hybrid samples, MFC gel was first dispersed in 40 g of water, as explained in Section 4.1, then this aqueous solution along with the rest of water required for preparing the concrete mix was used to treat RCSF following the process described in Section 4.2. After adding water/aqueous mix to the solid mix, this was mixed with an electrical drill for 5 min. Prior to pouring the mixtures in the mold, a very thin layer of hydraulic oil was applied to the internal surface of the molds to prevent adherence between the mixture and mold. The molds containing mixture were vibrated on a vibrator for 2 min to remove any air bubbles. After preparing the mixtures, the molds were left untouched for one day to allow the specimens to harden. Once samples had been extracted from the molds, the specimens were placed in a curing tank filled with water for the curing process for 28 days.

### 4.4. Experimental Testing Program

#### 4.4.1. Slump

The slump test was carried out on fresh concrete mixes to evaluate the effects of RCSF and MFC gel on the consistency and flowability of the concrete, following the BS EN 12350—2:2019 standard [62]. The test utilized a plastic Abrams cone with a height of 150 mm, a top diameter of 45 mm, and a base diameter of 95 mm. After pouring the fresh mixture inside a cone, the cone was compacted with vibration for 10 s and its top surface was leveled. Subsequently, the cone was gently lifted in a vertical direction without any lateral or torsional movement. The slump was then measured as the vertical difference between the top of the cone and the highest point on the deformed concrete surface. Then the slump index was calculated using Formula (1):

(1) $$Slump\;index=\frac{\left(Initial\;heigth-Final\;height\right)}{Final\;height}$$

#### 4.4.2. Three-Point Flexural Strength

The flexural strength testing was conducted using a Zwick-Roell Z10 universal testing machine (Zwick-Roell GmbH & C. KG, Ulm, Germany) with a 10 kN load cell. The test was performed in a three-point configuration with a 100 mm span length, in accordance with the ASTM C348 [63] standard test method. The preload was set to 0.1 MPa and the test speed was set to 1 mm/min. TestXpert computer-controlled testing software was used for the selection of test parameters and for data acquisition.

For each formulation, three beams of approximately 160 mm × 40 mm × 40 mm were tested. The results of the flexural test were expressed in terms of flexural strength. The average values of flexural strengths, along with their standard deviations, were calculated.

#### 4.4.3. Compression Test

The compression test was conducted on a 40 mm per side cube specimens extracted from broken beams from the flexural tests, following the requirements of ASTM C109/C109M-21 [64]. The Zwick-Roell Z150 testing system with a 150 kN load cell was used for the test. The preload was set at 1 MPa, and the test speed was 1 mm/min.

The results of the compression test were expressed in terms of compressive strength. Four specimens were tested for each formulation, and the average values and compressive strengths, along with their standard deviations, were calculated.

#### 4.4.4. Fracture Surface Observation

Fracture surface observation is a valuable technique for analyzing the behavior of materials under stress. By examining the surface of a broken specimen, we can gain insight into the macrostructure and mechanisms that led to its failure. In this case, the fracture surface of three different families of specimens were observed, to investigate the effect of each additive. The failure patterns were acquired with a Canon Powershot SX210 IS (Canon Inc., Tokyo, Japan) digital camera.

#### 4.4.5. Scanning Electron Microscopy (SEM)

Scanning electron microscopy (SEM) investigation was conducted using a Tescan MIRA 3 to analyze fiber deagglomeration, fiber–matrix interaction, void size/quantity, and fiber reinforcement effects (pull-out and crack bridging). Sample sizes around 5 mm × 5 mm × 5 mm for each type of formulation were collected from central parts of fractured specimens after the compression tests and gold coated using an Edwards sputter-coater S150B (Edwards Ltd., Burgess Hill, UK).

#### 4.4.6. Water Absorption and Porosity

In order to measure the cementitious samples’ water absorption (WA) values, a modified ASTM C1585-13 standard [65] was employed. Based on the procedure by El-Seidy et al. [66], the samples with various formulations were cut into cubic shapes with (40 × 40 × 40) mm^3^ dimensions. Afterward, these cubic samples were dried in an air oven at 60 °C for 24 h. First, the specimens were weighed (M_0_) and then placed in a water container. From their fracture surface, the samples were in contact with the water; the water level in the container was 5 mm, and this was kept constant during the test. At specific and different time intervals after to the immersion, the soaked samples were removed from the container, excess water was blotted off with a towel, and the samples then weighed again (M_t_). The selected time intervals were 5 min, 10 min, 15 min, and 30 min, and 1, 2, 4, 6, 8, 24, 48, and 72 h. According to Equation (2) below, the average WA was calculated. For each sample composition, two samples were analyzed for WA media as explained:

(2) $$WA\%=\frac{\left(M_{t}-M_{0}\right)}{M_{0}}\times 100$$

A vacuum saturation technique (ASTM C1202-19 Standard [67]) was implemented to assess the permeable porosity of the concrete-based samples. Figure 6 illustrates the experiment apparatus. Cubic samples, with a 1 × 1 cm^2^ surface area, were prepared and weighed (M_0_). Beforehand, these cementitious specimens were dried in an air oven at 60 °C for 24 h. In the next step, they all were put in an empty glass vacuum desiccator connected to a LABOPORT N 86 KN.18 Mini Diaphragm Vacuum Pump (KNF Neuberger GmbH, Freiburg, Germany) with a 6 L/min flow. For 30 min, the desiccator was evacuated by pumping to reach a 0.5–0.6 bar vacuum level. As the vacuum level stabilized, the samples were kept under the same condition for another 5 min, and then the pump was switched off for 2 min. This cycle was repeated five times. After that, the desiccator was filled with tap water to cover the concrete cubes, and again, the air inside was evacuated for a further 30 min to obtain a 0.5–0.6 bar vacuum level. The samples were preserved under pressure for 5 min; after that, the pump was tuned off for 2 min. This protocol was repeated five times. Last, a METTLER TOLEDO hydrostatic balance was utilized to identify the mass of the saturated specimens in air (M_a_) and the soaked specimens in water (M_w_). As seen in Equation (3), the average porosity percentage (%) was computed. For each formulation, three cubes were analyzed:

(3) $$Porosity\%=\frac{\left(M_{a}-M_{0}\right)}{\left(M_{a}-M_{w}\right)}\times 100$$

## Figures and Tables

**Table 1:** Mix proportion of mixtures.

Sample ID	Fiber Percentage	RCSF (kg/m^3^)	MFC (kg/m^3^)	ANC (kg/m^3^)	Cement (kg/m^3^)	Sand (kg/m^3^)	Water (kg/m^3^)
CTR	-	-	-	-	700	1170	295
0.5R	0.5% RCSF	3.5	-	3.5	700	1170	297
0.75R	0.75% RCSF	5.25	-	5.25	700	1170	298
1R	1% RCSF	7	-	7	700	1170	300
0.5M	0.5% MFC	-	1.48	-	700	1170	295
0.75M	0.75% MFC	-	2.21	-	700	1170	295
1M	1% MFC	-	2.95	-	700	1170	295
0.5RM	0.75% MFC + 0.5% RCSF	3.5	1.48	3.5	700	1170	297
0.75RM	0.75% MFC + 0.75% RCSF	5.25	2.21	5.25	700	1170	298
1RM	0.75% MFC + 1% RCSF	7	2.95	7	700	1170	300

## Data Availability

The data presented in this study are openly available in the article.
